# Exploitation of puddles for breakthroughs in claustrum research

**DOI:** 10.3389/fnsys.2014.00078

**Published:** 2014-05-14

**Authors:** John-Irwin Johnson, Brian A. Fenske, Amar S. Jaswa, John A. Morris

**Affiliations:** ^1^Division of Anatomy, Department of Radiology-Anatomy, Michigan State UniversityEast Lansing, MI, USA; ^2^Neuroscience Program, Michigan State UniversityEast Lansing, MI, USA; ^3^SNBL USA, Ltd.Everett, WA, USA

**Keywords:** minipig, mammal brain diversity, proteomic labeling, multiprobe microelectrode arrays, large mammal gene expression studies

## Abstract

Since its first identification as a thin strip of gray matter enclosed between stretches of neighboring fiber bundles, the claustrum has been considered impossible to study by many modern techniques that need a certain roominess of tissue for their application. Known as the front wall, *vormauren* in German from 1822, and still called *avant-mur* in French, we here propose a means for breaking into and through this wall, by utilizing the instances where the claustral tissue itself has broken free into more spacious dimensions. This has occurred several times in the evolution of modern mammals, and all that needs be done is to exploit these natural expansions in order to take advantage of a great panoply of technological advances now at our disposal. So here we review the kinds of breakout “puddles” that await productive exploitation, to bring our knowledge of structure and function up to the level enjoyed for other more accessible regions of the brain.

## What is a puddle?

The simplest and canonical notion of the claustrum, dating from its discovery by Vicq d'Azyr (1786), has it a thin subcortical layer of gray matter extending from superior to inferior in the white matter between the insular cerebral cortex and the underlying putamen. Looking a bit further, virtually all mammalian claustrums do indeed show such a lamina, and at one point in its inferior reach, the layer curls around the fundus of the rhinal sulcus that separates insular and other meso- and neocortex above from the olfactory allocortex below.

In their seminal 2009 work, Mathur, et al. detail many of the details of the difficulties encountered in attempts to learn the functioning and organization of a thin laminar entity, and the limitations this has posed on learning much about what the claustrum is and does. In this article we have a pregnant suggestion about how to overcome this inherent difficulty, by utilizing natural biodiversity that is seen in the claustrum in a variety of species.

The curling claustral lamina descends beneath the rhinal sulcus fundus then spreads out a bit into a more diffuse group of subcortical cells internal to those of the trilaminar olfactory allocortex that has become known as the endopiriform region or “nucleus.” This bridge from suprarhinal to infrarhinal regions shows no consistent break or marker between the claustral and endopiriform cell groups. We will hereafter term this consistent connection, frequent among the mammals that we have observed, the “root of the claustrum” and it may indeed be the region from which cells have migrated in opposite directions to grow into the adult claustrum and endopiriform regions.

### Superior pyramidoid puddles

**Figure 1:**
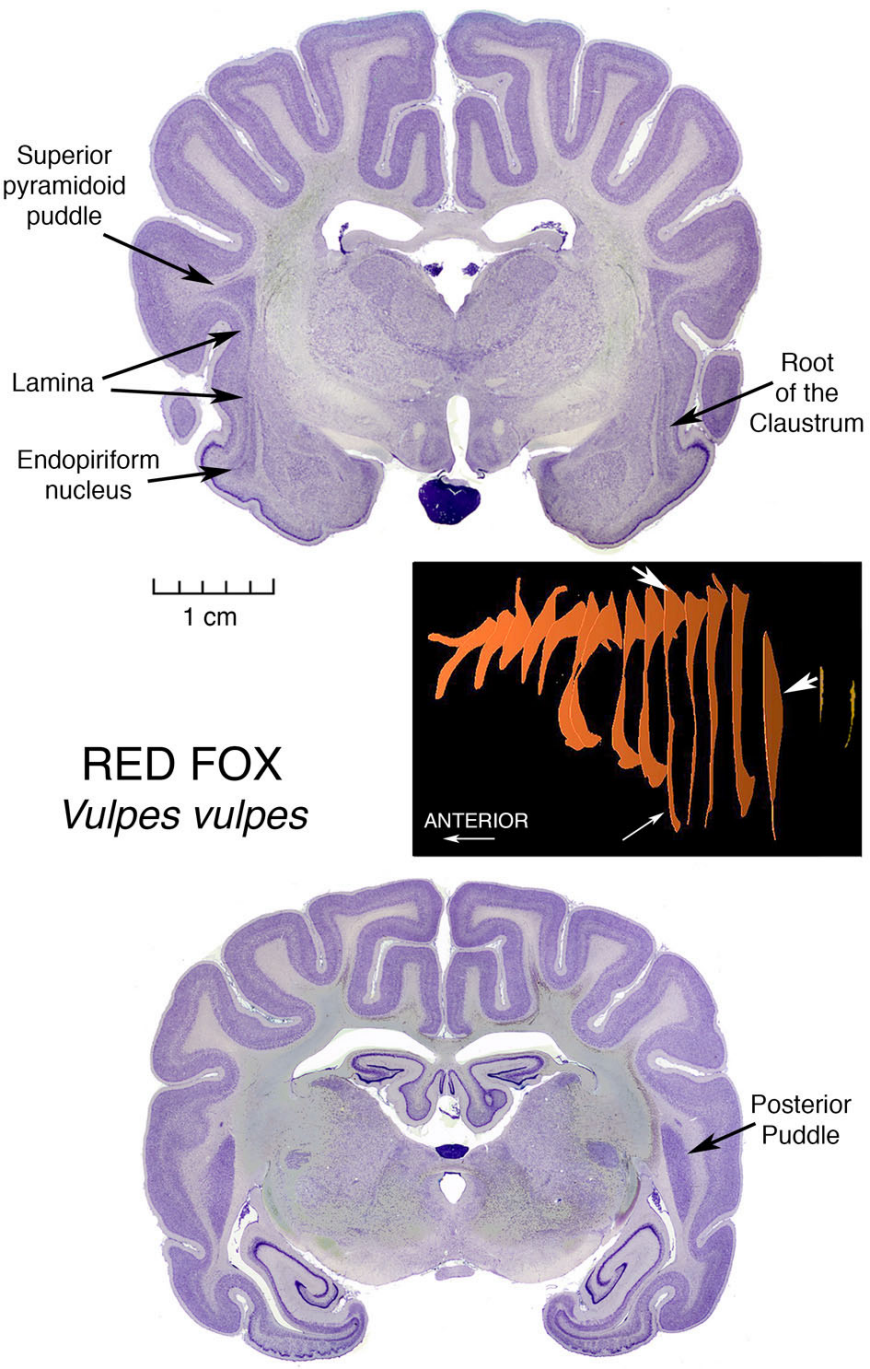
**Nissl stained coronal sections through the brain of a Red Fox, illustrating the morphology of the Superior Pyramidoid Puddle, the Root of the Claustrum, the Endopiriform Nucleus and the Posterior Puddle**. Accompanying these histological images is a three-dimensional diagram, constructed from outlines of the Claustrum in these sections in series with those of other sections forming an evenly spaced sampling of the entire claustrum. The reconstructions were obtained using the ImageJ program. White arrows on the reconstruction denote the 3-dimensional localization of the features labeled in the section images by black arrows. The specimen in this Figure, as are those in all but the last Figure 7, is from the Welker Wisconsin Comparative Collection of Mammalian Brains, as scanned and displayed on the website at http://brainmuseum.org.

Figure 1 presents a clear illustration of this root region, with endopiriform below the sulcal fundus. And then, extending in a long thin and straight lamina, this claustrum of a Red Fox, *Vulpes vulpes*, spills out at its top, as seen in two dimensions, into an elegant triangular formation encompassing large accumulation of apparently claustral cells, in what we call the superior pyramidoid puddle. This graceful vulpine display introduces our notion of claustral puddles.

The puddle fills the wider space occasioned by the ending of the top of the putamen, which at anterior and lower levels may block the expansion of the claustrum medialward, and laterally by the folding of cerebral cortex opening a space in the middle of the fold, allowing the claustrum to “invade” the core of the gyrus. The space is bounded laterally by U fibers traveling between the tops of the neighboring gyri and accounting for most of the triangular appearance of the cell mass. This invasion, by points of claustrum entering cores of overlying gyri, was first depicted by Vicq d'Azyr (1786) in the first published description of a claustrum, and was shown in a human brain. This remarkable painting can be viewed directly at http://www2.biusante.parisdescartes.fr/livanc/?p=22&cote=00519x02&do=page We will see this gyrus invasion phenomenon repeated in many diverse animals.

At the posterior end of the fox claustrum, there is another enlarged region, constituting a Posterior Puddle, and we will take up that topic after further consideration of Superior Pyramidoid Puddles.

#### A superior pyramidoid puddle has been the site of a major breakthrough in understanding claustral structure and function

In the domestic cat, a superior pyramidoid puddle was the occasion of a major advance in the understanding of structure and function of the claustrum, in several articles by LeVay and Sherk (LeVay and Sherk, 1981a,b; Sherk and LeVay, 1981a,b, 1983; LeVay, 1986), and additional contributions by Olson and Graybiel (1980), and by Irvine and Brugge (1980). This series of studies was made possible by the wider expanse of the puddle compared with the lamina, enabling the location of arrays of projections inside the claustrum in somatotopic order. Analysis of the posterior end of this region of claustral expansion, using a coordinated panoply of anatomical and physiological techniques, yielded the knowledge that this restricted claustral region has extensive reciprocal connections with cortical sensory association regions in visual, somatic sensory, and auditory pathways, and suggested the concept well expressed by Olson and Graybiel (1980), that claustrum functions as something of a servicing satellite aiding these cortical regions in the dynamic regulation of receptive field properties of somatotopically organized sensory circuits in cerebral cortex. Earlier findings by Graybiel (1978), and Sherk (1979) had suggested that the small parabigeminal nucleus in the midbrain served the much larger superior colliculi as just such a satellite processor.

Of further interest is that these three claustral sensory regions are located in somatotopic parallel with the arrangement of their corresponding (in many senses of the word) cortical areas, with the visual area caudalmost, the somatic sensory area superiormost and the auditory area inferiormost. Furthermore, just as the cortical areas are located in roughly the posterior region of the hemisphere, while motor regions are in the anterior portion, so the posterior location of the sensory regions leads to speculation that the substantial more anterior remainder of this great puddle is devoted to relationships with motor cortical regions.

A somewhat similar arrangement is seen in the larger cortical-reciprocally-active brain part: the dorsal thalamus.

Similar superior pyramidoid puddles (see Figure 3) are seen in other, but not all, carnivores, as well as in perissodactyl zebras and artiodactyl llamas, zebus (representative of cattle or oxen), and pigs (Buchanan and Johnson, 2011).

### Posterior puddles

#### A giant among puddles, and among research opportunities?

But in the pig, proceeding caudalward, the pyramid rather suddenly explodes into the “mother of all” puddles, a giant oblong mass of claustral tissue that separates into parallel lobules for a considerable distance caudalwards. This giant posterior puddle is a prime candidate for contemporary investigations. It will accommodate large injections that will remain completely within the claustrum, and multiprobe microelectrode arrays for recording simultaneously from several different intraclaustral active loci. It will provide large numbers of neurons, interneurons, possibly glia and other structures for quantitative assessments without contamination from neighboring fiber bundles. Here can be large concentrations of claustral neurotransmitters, and large populations of receptive synapses.

The dimensions of the puddle render possible the advantages of punch techniques for determining levels of chemical contents, and isotropic optical fractionation methods for cell counting (Herculano-Houzel and Lent, 2005).

Furthermore, small varieties of this species of artiodactyl are being well-developed as convenient and informative subjects for mammalian biological experimentation. That they have large and complex gyrencephalic brains adds to their potential for studies of functions that may not exist in small, simple, and perhaps vestigial brain regions in the murine rodent populations that have become the overwhelmingly dominant subject population at this time in the course of neuroscientific research. It is of particular interest that the same four lobules appear in a figure from a section of a minipig brain (Guidi et al., 2011, p. 173 Figure 2 Frame 17.28), published to show something quite different—it was quite by chance that this section occurred right at the lobulating point in the minipig Posterior Puddle, leading to speculation that there could be functional significance to this peculiar organization of claustral tissue, such that it is replicated in individuals of very different size. That such a local feature is reproduced from individual to individual marks this puddle as an opportunity to study local organization within the claustral tissue.

**Figure 2:**
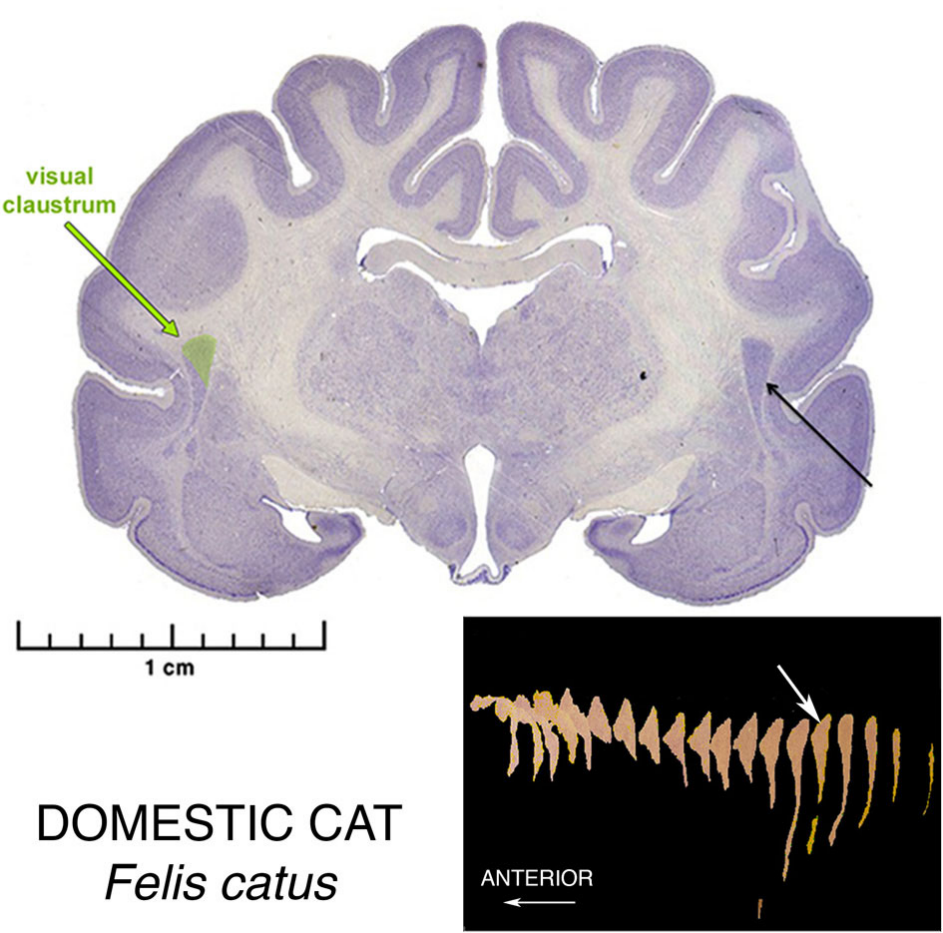
**Nissl stained coronal section the brain of a Domestic Cat, illustrating the morphology of the Superior Pyramidal Puddle**. Within the puddle, the green color shows the location of the Visual Sensory Area of the claustrum. The Somatosensory Area is located just rostral, and the Auditory Area just rostro-inferior to the Visual Area. The Visual Area is the largest of the three. The white arrow in the three-dimensional reconstructions shows the location of this section in the whole claustrum. These sensory areas are found in only the posteriormost part of this Puddle.

**Figure 3:**
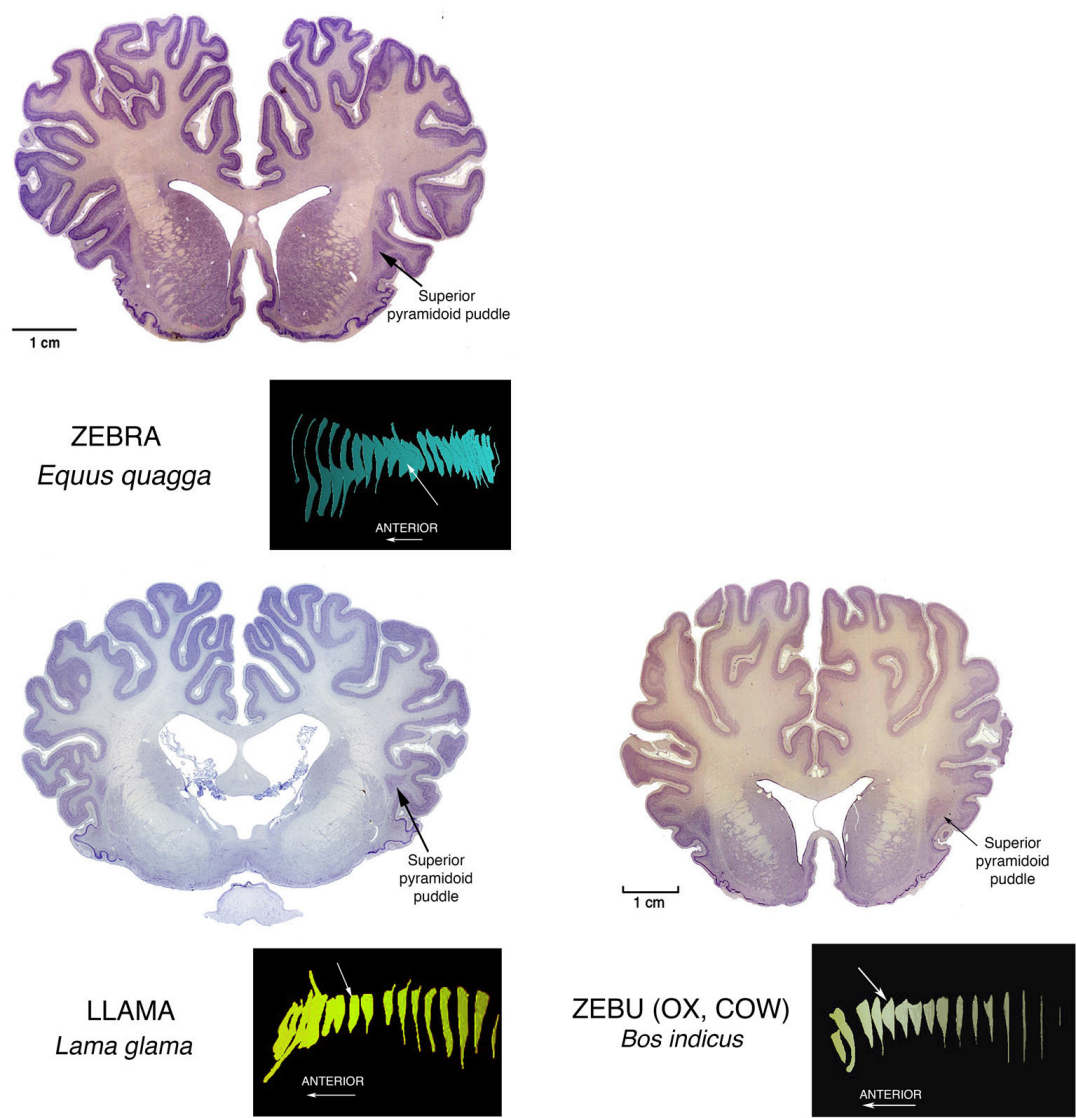
**Nissl stained coronal sections through the brains of perissodactyl Zebra, and artiodactyl Llama and Zebu (Ox, Cow) illustrating other instances of Superior Pyramidoid puddles**. The white arrows in the reconstructions mark the positions of the illustrated sections in the anterior-posterior extents of the claustrums. This puddle shape along the superior aspect of the claustrum can occur anywhere from front to back. Its appearance may depend more upon the proximity of a relatively open overlying gyral core rather than to any consistent functional or genetic reason. This in turn may mean that the claustral mass can assume any convenient shape, and this in turn suggests that internal connections inside the claustrum are rare, and not of general functional significance.

**Figure 4:**
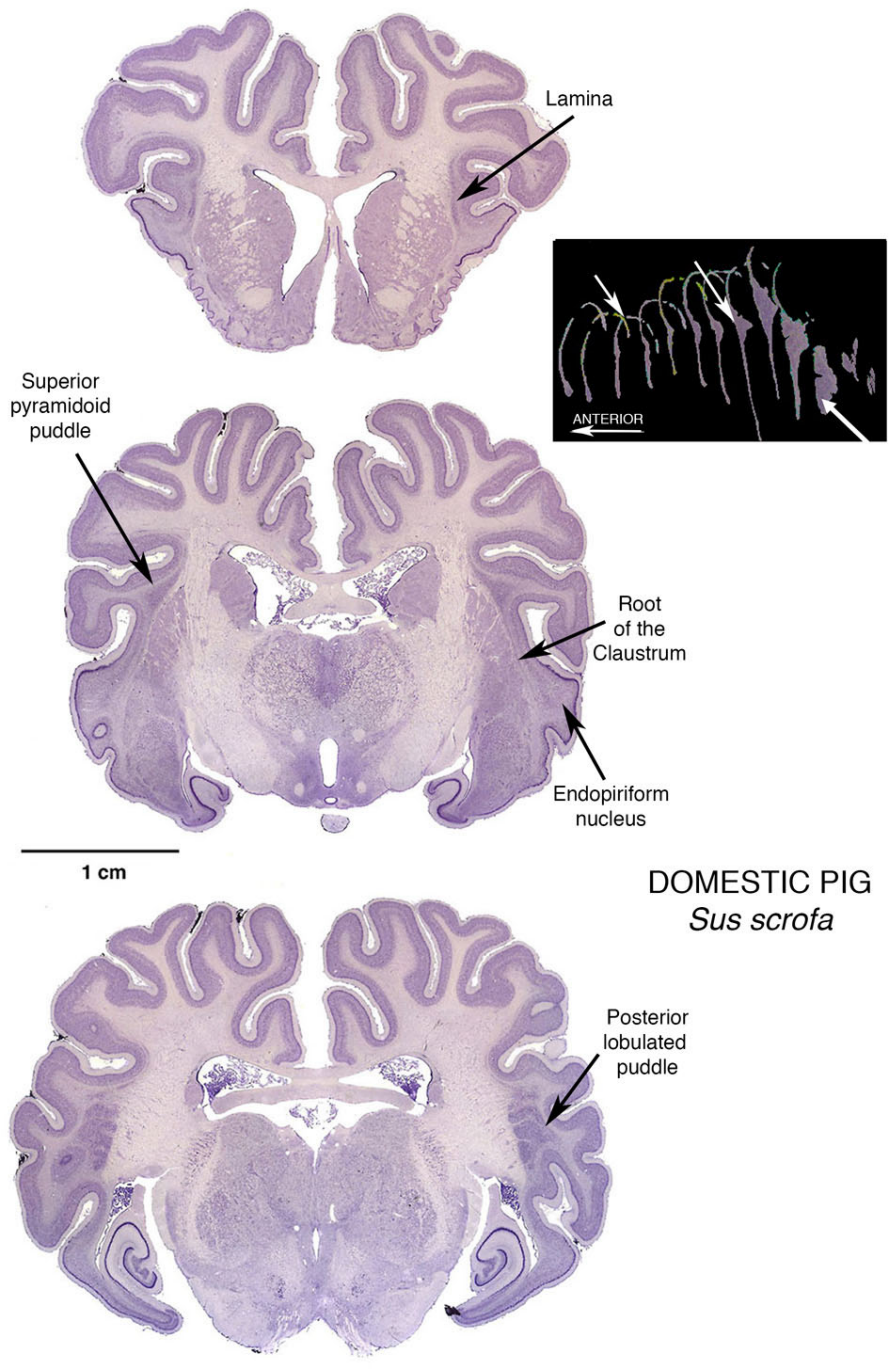
**Three Nissl stained coronal sections through the brain of a Domestic Pig, showing a modest Superior Pyramidoid Puddle that abruptly expands, posterior to the caudal limit of the putamen, to form a very large Posterior Puddle**. This puddle, seen in this specimen and other published sections from other pigs, has a distinct lobulations into what appear to be four bars of cells, more or less separated from one another. This interesting arrangement is just one of many reasons for further investigation: if the claustral mass is sufficiently large, are there internal structural or functional reasons for assuming a particular shape? The 3-dimensional reconstruction in this figure is by Kenneth Buchanan.

As a further bonus, as seen in the middle section in Figure 4, pigs possess the “mother of all” endopiriform regions, an unusually large and homogeneous region underpinning an expansive olfactory cortex that actually becomes gyrencephalic, folding its expanse into the space available in the front of the brain case. These animals thus become subjects of choice in an additional challenging task: determining just what is the endopiriform nuclear region and what does it do?

This becomes particularly important with the disappearance of the traditional cats and dogs from experimental laboratories. The current fad that permits only small murine rodents as suitable experimental subjects may be reaching its rate-limiting condition in brain study. Tiny brains can reveal only so much that is relevant to the operations of large brains, and the pigs with their large enough brains, and particularly available claustrums (and endopiriform too as a fortunate lagniappe) may well represent the avenue to rapid advancement in the study of claustrum and related structures. The olfactory cortex in pigs is unusual in that it has gyri and sulci, and the endopiriform cells extend up into the core of the upper gyrus, much as the claustral cells extend into the white matter of insular and other neocortical and mesocortical regions.

### Inferior puddles

#### The inferior puddles of anthropoid primates

**Figure 5:**
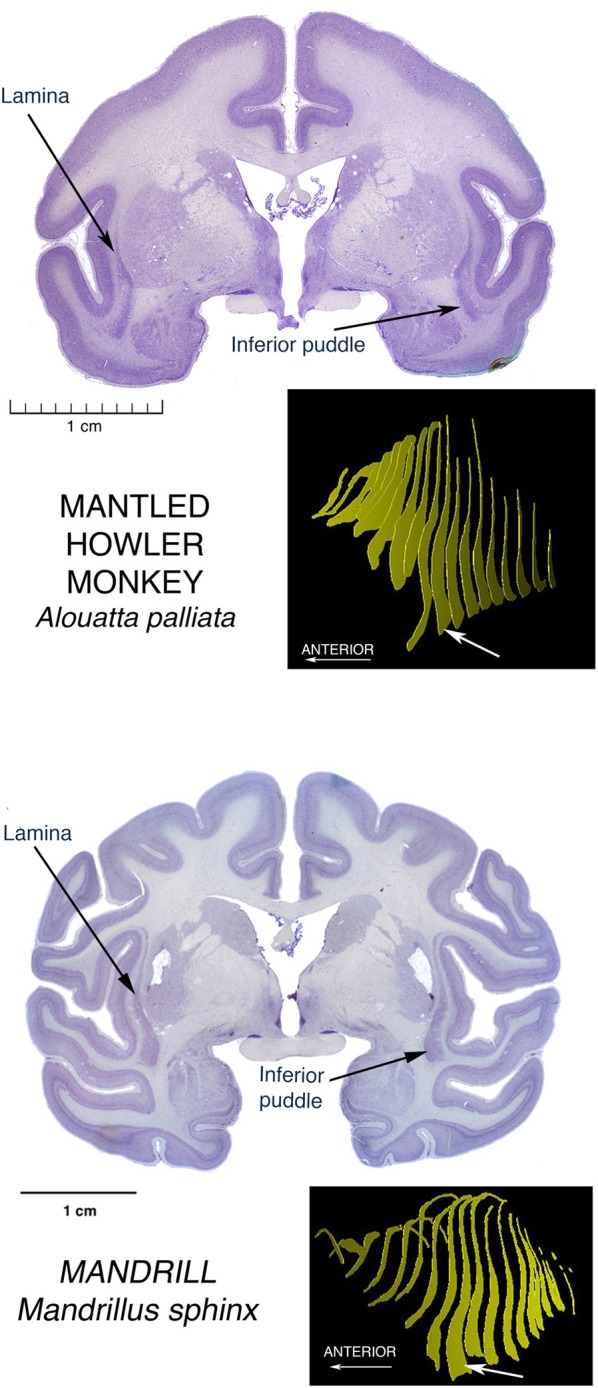
**Nissl stained coronal sections through the brains of two anthropoid primates, showing the Inferior Puddle seen in all members of this group that have been investigated, including humans**. The New World Platyrrhine monkeys are represented here by the largest of these species, the Mantled Howler Monkey. The other major monkey radiation, the Old World Catarrhine monkeys, is represented here by the brain of a Mandrill.

Another large puddle of particular interest, since we humans possess this one, is the large Inferior Puddle seen in anthropoid primates. In the array in Figure 5 it can be seen as a prominent and constant feature in the largest of the New World platyrrhines, the Mantled Howler monkey, and representing the Old World catarrhines, a Mandrill.

On the cover of the carefully detailed human brain atlas of Mai et al. (2008), and also on pp. 127–153, the Inferior Puddle, prominent and in the standard shape, contains five different subdivisions of the claustrum.

See the article by Baizer in this issue, to see the Gorilla version of the Inferior puddle.

In a macaque, Ribeiro Gomes et al. (2013) showed in a poster at the 2013 meetings of the Society for Neuroscience, early results of an extensive study identifying regions of claustrum with reciprocal connections with contrasting pairs of restricted regions of cerebral cortex. Regions were selected to be far from one another both spatially and functionally. Most of the cortical regions were connected with a stripe of claustral cells running for some distance across the diagonal extent of most of the claustrum, much as Mathur (2008) described for the cortical connections of a local region of cortex in a stripe of claustral cells running across the whole claustrum in rats. But in one case, a region of visual association area, V4, had its connections instead with a tight ball of cells located right in the center of the inferior puddle. To have an occipital region of cortex connected to the inferiormost extension of claustrum may point to a specialization of the puddle as something different than the usual long stripe of connections with a particular cortical area.

### Anterior puddles

#### Prominent anterior puddles in marsupials

**Figure 6:**
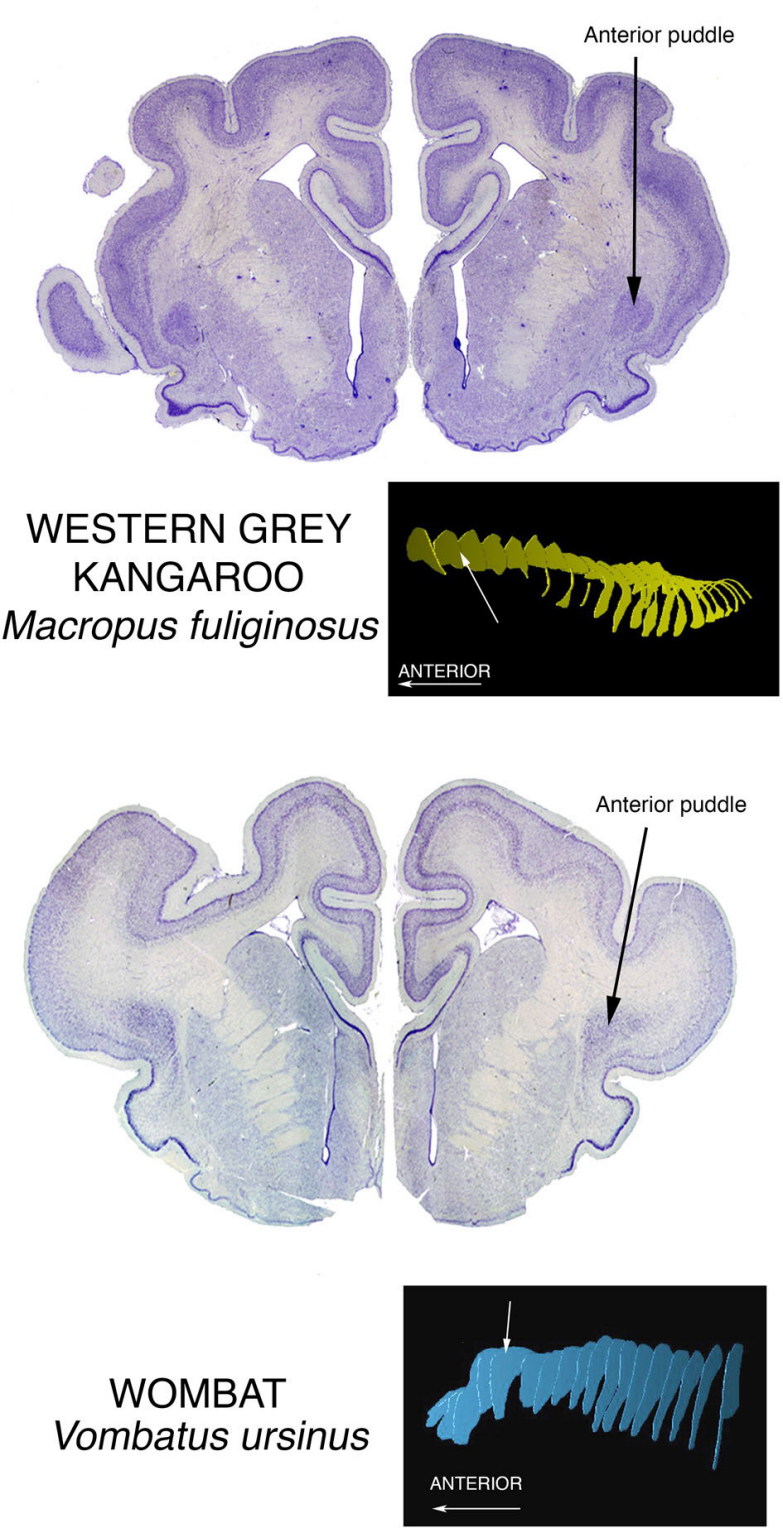
**Nissl stained coronal sections through the brains of two large marsupials show the Anterior Puddle seen in all marsupials of whatever body size**.

We have seen superior, posterior, and inferior puddles thus far. To complete the spatial possibilities, there are the large anterior puddles seen in all of the marsupials we have examined. They are shown in the brain of a wombat and a kangaroo, two of the largest of marsupial brains, in Figure 6. Very small marsupials also exhibit them, see them in brains of bandicoots and antechinuses. This taxon-wide commonality could be related to the arrangement of interhemispheric connections in marsupials. In place of a corpus callosum, the hemispheres communicate mainly through a greatly enlarged anterior commissure, and this structure forms a wall beyond which claustral cells cannot go. Therefore, they pile up at the front end of the corpus striatum. This condition is also seen in anteaters and sloths of the order Xenarthra, although these animals do have the normal placental large corpus callosum.

## What is a claustrum?

Debate over categorization of cellular groupings in the brain likely began immediately after the second examination of a specimen. Nuclear groupings have commonly been sorted by descriptions of apparent shapes and location, resorting to nomenclatures that often use prepositional modifiers relative to the few unambiguous landmarks that are argued least. So the literature contains examples such as the posterodorsal medial amygdala which translates as the top, back part, of the part nearer the midline, of a grouping that exhibits a vaguely almond-like shape, which results from a human brain cut just so, presumably from a vantage of sufficient distance to highlight the subject with appropriate contrast given the ambient light source and viewer acuity. Such is the practical convention that largely supplanted regional designations from the name of an initial publicist; with a few exceptions such as the Nucleus of Darkschewitz, a name that may persist purely on enigmatic connotation.

Careful research and means of observation have come about that give hope to a functional nomenclature, often more behavioral than purely physiological, such as ascribing a portion of the cortex found to primarily be involved in motor function, as “motor cortex,” (and not “uniform high amplitude discharge zone”). Yet even there, consideration of various inputs and nearby areas of similar but not redundant function, results in a parcellation that yields primacy, where premotor and secondary motor areas then arise. Functional nomenclature is useful, but ultimately just another layer of metadata describing the brain, and not overly awkward, even when the motor cortex is idle. Of course regions of the brain do many different things together in varying concert, and the coordination and conduct of these areas must be measured to be understood.

Furthermore, the variation of brains is as immense as speciation, and even within a species, individual variance frustrates the logical desire to impose a Cartesian coordinate grid and retire the names. So in order to measure parts of the brain and communicate these findings, two questions must be answered together, both of which may vary considerable depending on definition; where precisely, are the limits of the claustrum, and what characteristics at its location are robust yet stable.

In the case of the claustrum, the convenience of being a bolt of cells enclosed by a barrier of white matter allows for a literal naming that serves as a unique reference, yet a lingering presumption is still implied, that the subject matter at hand is human or other sizable brain and sampled as its namesake, avoiding variability at the bulbous margins. It remains to be determined whether homogeneity of claustral elements is sufficient to keep its name.

### Direct proteomic marking

Mathur et al. (2009), have revolutionized conceptualization of what claustrum is and does, and they have done it using a fashionable small murine rodent. They did this by recognizing a first principle, that one must be able to identify claustral cells as such, and to this end developed a proteomic marking technique that specifically would identify claustral cells, particularly as contrasted with cells of cerebral cortex. Their results demonstrated, in the claustrum of rats, that the claustrum is really not a different place than cortex, but rather a region that has a number of cells that are different from the standard cells of cerebral cortex, particularly in their shape and arrangement relative to their neighbors. It is this distinctive shape and arrangement that led early debaters, chiefly Santiago Ramón y Cajal (1900), to decide that claustrum is a different kind of entity rather than just being another layer within or adjacent to cortex as proposed by, prominently, Meynert (1885) and Brodmann (1909).

Mathur et al. (2009) demonstrated that both opinions are true, but that the distinctive claustral cells in fact are intermingled with, and submerged into, a layer of cortex.

A major question now demanding resolution is whether this situation of submersion in cortex is a general feature of all claustrums, or is it a condition of a regressive vestige of a once robust claustrum in a species that is divesting itself of claustral function in the course of its evolution, just as arboreal primates jettisoned most of their olfactory systems in the course of their evolutionary adaptations.

We believe that a great deal can be learned by application of current proteomic identification techniques to each of the major puddles that we have described, beginning with the giant posterior puddle of the pig. This will provide almost instant answers to the questions whether the Mathur et al. picture of the claustrum as an internal cortical mechanism represents a general depiction of claustrum structure and function across mammals, or is it a portrait of a degenerate structure on the point of disappearing from the brain mechanisms of the small murine rodents?

One currently popular indirect method of proteomic identification is the use of markers of gene expression.

### Studies of gene expression. what can the genes reveal about the claustrum and the puddles? what can the claustrum and the puddles reveal about the genes?

Fortunately, a level of study that both controls for and accepts variation may yield understanding that neither attempt could achieve alone. In the laboratory, size, fecundity, and hardiness are the essential requirements that led to the selection of rodents as a research model. Substantial inbreeding allowed for the control of genetic variation, and hence brain variation, but the fortuitous susceptibility of mice to gene manipulation drove the consolidation of research, in only two decades, decisively to this species. Such control over genetic variation allows for precise measures of cellular organization and functional localization. This has provided for surprising discoveries that would have been lost, or not even considered, due to background variation in neural characteristics.

For example, because of their olfactory centered ethology and diminutive brain size, mice were overlooked as a model for the study of vision. Yet the anatomical precision afforded by consistency in genotype, quickly allowed for a number of subdivisions to be uncovered in the mouse visual cortex based on cortico-cortico connections and cytoarchitecture revealed by neurofilament protein, first by Wang and Burkhalter (2007) and van der Gucht et al. (2007), and then the subsequent functional verification of these subdivisions by Marshel et al. (2011). Similarly, gene expression can guide further studies of function by showing where brain areas subtly differ. The claustrum puddles in different locations by species, but if it does not in mouse, it could be due to an egress toward a “vestigial” state, or alternatively because it has not been examined closely.

#### An exploration of gene expression patterns

To more closely examine the claustrum of *Mus musculus* C57/B6 mouse (hereafter called just mouse) using gene expression as the categorical metric, we conducted gene expression experiments. For this we used a published resource presenting gene expression experiments for all brain areas and all known genes, from the Allen Brain Atlas (Lein et al., 2007) (brain-map.org). These are made available online for public interrogation. We examined these experiments in a non-systematic way for gene expression patterns around the adult male mouse claustrum in order to inform whether claustrum (a) could be isolated from the deepest layers of cortex, (b) disambiguated from an endopiriform region, and (c) separated into a shell and core or beyond. These are questions that will persist until more data is compiled. It may be that protein expression, electrophysiological characteristics, cellular morphology, or cell type composition can distinguish these features in this region of the brain. In our case, we used gene expression to test these suggestions and compare findings.

The overall prediction was that gene expression experiments would confirm previous findings, but that careful attention could reveal subtle distinctions in the mouse brain, which might then lead to similar findings in a primate brain. Expected as well was organizational conformation in general between the species, but it would be good to uncover any exceptions that reveal species differences in gene expression or structural composition, or both. Therefore, we concurrently searched the Allen Atlas subset of gene expression experiments in the brain of the monkey *Macaca mulatta* (hereafter called macaque) for similar features, including differentiation of the Inferior Puddle of the claustrum.

Differential expression patterns found in mouse were compared in macaque where available. Our list included *Nr4a2* (nuclear receptor subfamily 4, group A, member 2), *Ntng2* (netrin G2), *Synpr* (synaptoporin), *Ctgf* (connective tissue growth factor), *Rgs4* (regulator of G-protein signaling 4), *Slc17a8* (solute carrier family 17 (sodium-dependent inorganic phosphate cotransporter, member 8), *Crym* (crystalline, mu), and *Rorb* (RAR-related orphan receptor beta).

**Figure 7:**
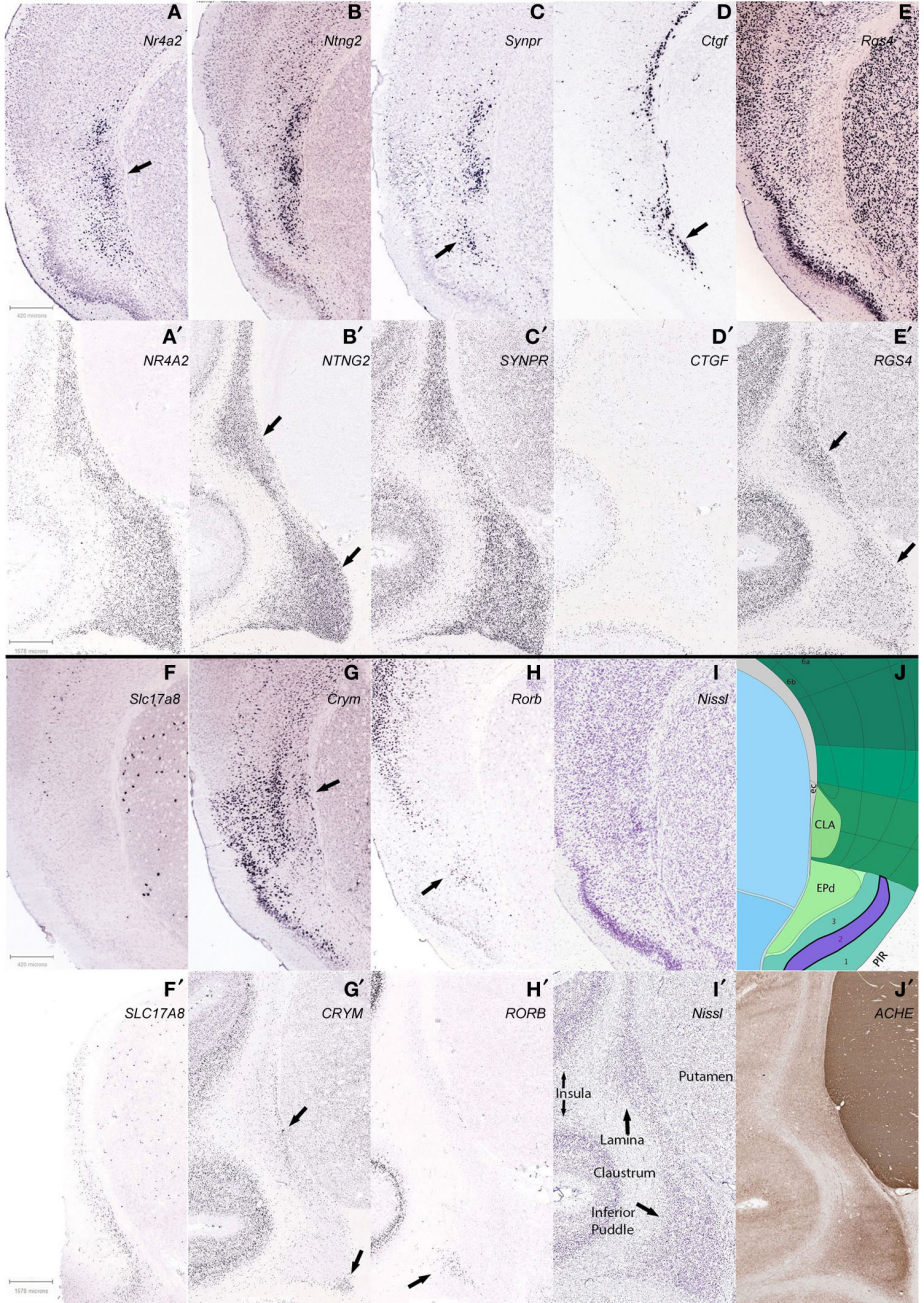
**Differential claustrum expression patterns within and between species, C57/B6 mice in 1st and 3rd panels (A–J), Macaque monkey *Macaca mulatta* in 2nd and 4th panels (A′–J′). (A)** *Nr4a2* expression forms a dense core (arrow) surrounded by diffuse staining, with another dorsal cluster that is also found with *Ntng2* and *Synpr* labeling **(B,C)** and reciprocally in *Crym* **(G)**. **(D,D′)** *Ctgf* labeling of cortical layer 6b and deeper endopiriform nucleus (EPd), with a stream of cells that subtend the claustrum in mouse (arrow), and only deep cortex and non-neuronal appearing cells lightly labeled in macaque. **(E,E′)** *Rgs4* labeling avoids the mouse claustrum yet labels most other regions, but staining in macaque shows a species difference. **(F,F′)** *Slc17a8* shows another stark species difference in claustral expression, with no apparent expression in mouse and many cells expressing in macaque. **(G,G′)** *Crym* expression in mouse largely avoids the claustrum but the arrow shows a wedge of cells that together with the more superficial labeling, surrounds the claustrum, while in the macaque labeling also avoids the claustrum, but reveals a deep stream of cells in the lamina and the deep corner of the inferior puddle (arrows). **(H,H′)** Arrows show that *Rorb* expression marks the superficial EPd in mouse, and labels only the gyral portion of the inferior puddle in macaque. **(B′,E′)** Apparent reciprocal expression of *NTNG2* and *RGS4* in the macaque (arrows), in particular the inferior puddle. All macaque sections were from the same specimen except **(F′)**, and mouse experiments were from different specimens. Gene names are indicated (top right) of panels. See text for gene names. **(I,J)** Matching Nissl stain and atlas plate from Allen Mouse Brain atlas. **(I′,J′)** Two representative sections from the macaque brain are shown, stained for Nissl and acetylcholinesterase, respectively.

***Our findings. Segregation of brain subdivisions***. We found the mouse claustrum to be compartmentalized loosely into a core and shell pattern, as suggested by previous studies (Dávila et al., 2005; Legaz et al., 2005). Genes were found that show expression, or lack thereof, mostly within the Nissl-defined claustrum of the Allen Reference Atlas, in a discrete, dense pocket of cells. The most distinctive of these (*Nr4a2*, *Ntng2*, *Synpr*) showed positive expression in a dense core of the claustrum (Figures 7A–C). This is distinctly reminiscent of the Mathur et al. (2009) findings, in rats, of a bolt of claustrum cells ensconced in a “shell” of cortical cells, which also appear different from cortex.

In addition to the shell-core, the separation of the mouse claustrum from deep cortical areas, in particular the insular and gustatory cortices, could be discerned from reciprocal expression patterns such as *Crym* and *Ntng2* (Figures 7G,B).

Genes specific to the endopiriform area were more difficult to identify, as expected, due to the more common sharing of expressing cells between regions. While it may be noted in other figures to show apparent differential expression from the deepest layer of piriform cortex, layer 3, it is difficult to show a clear distinction from the claustrum, with the exception of *Rorb* (Figure 7H) that labeled a possible more superficial aspect, and *Ctgf* (Figure 7D) that appeared biased toward a deeper aspect of the endopiriform nucleus. Overlapping expression patterns enriched or diminished in each respective area could reveal on average a separation, but the evidence presented is that the two areas show variation, but substantial comingling of cells.

***Findings between genes***. We then examined expression patterns that discriminated the mouse claustrum from the deepest cortical layer, 6b, and found that *Ctgf* and *Crym* reveal the claustrum, if slightly separating it from the external capsule (Figures 7D,G). Likewise, positive expression of *Nr4a2* in the claustrum was found that seemingly avoided marking layer 6b in the claustrum (Figure 7A), while marking 6b for cortex in general. Another observation arose from 6b positive expression of *Ctgf* that revealed deep cortex underlying the claustrum, but then continued to reveal enrichment of the endopiriform area (Figure 7D). Further work will be required to characterize the cells underneath the claustrum, if such results are repeated in additional genes. The expression patterns examined revealed the claustrum concurrent with several different cortical patterns, in that definition of the claustrum coincided with restricted cortical layer 6b (*Nr4a2*), 6a (*Ntng2*), and a general scattered pattern across all layers (*Synpr*) as seen in Figures 7A–C, respectively. Cells that expressed clearly in all layers but 6b of cortex, avoided the claustrum in the case of *Rgs4* expression.

In the macaque claustrum, while gene expression largely occurred throughout the extent of the claustrum along with deeper cortical expression, a few expression patterns suggested heterogeneity, both within and between the lamina and inferior puddle. The deepest region of the lamina that abuts the putamen at the external capsule, appeared differentially enriched with *RGS4* expression, yet less so with *NTNG2* expression. Moreover, the opposite pattern was found in the inferior puddle, with *NGNG2* revealing it along with the superficial lamina, while *RGS4* labeled less intensely in the puddle and superficial lamina (Figures 7B′,E′, respectively). The inferior puddle was compartmentalized by *CRYM* expression in the deepest corner, along with the deep lamina (Figure 7G′). *RORB* labeled the superficial gyral corner of the inferior puddle only, without notable expression in the remaining extent of the claustrum.

Our data indicates that gene expression reveals differential expression within the claustrum, suggesting various developmental backgrounds and functions. As an example, the claustrum of both species is not labeled by *Ctgf* expression, yet the deepest nearby cortex shows strong labeling. That the deepest cortical layer of the mouse, 6b (layer VII), is quite compact whereas in the macaque labeling appears more broadly in layer 6, may be understood as an effect of development, where differential rates and periods between species of sub-plate apoptosis and white matter encroachment, produce a more distributed appearance in the primate (Reep, 2000). That same hypothesis also implies that various parts of cortex may experience differing developmental effects, as suggested by *Nr4a2* expression in layer 6b of mouse that, unlike *Ctgf*, diminishes or disappears under the claustrum. Whether these neighboring cells are homologous requires additional examination and instances, and subtle pattern differences in deepest monkey cortex do present intrigue. The inferior puddle also showed some interesting variance at the margins, but likewise will require additional data to fully uncover a consistent pattern.

***Findings between species***. Species differences were noted in many expression patterns across genes. The most conspicuous, *Slc17a8*, was completely absent in the mouse claustrum, while appearing throughout the macaque claustrum; yet the putamen of both species showed a similar pattern (Figures 7F,F′). *Ctgf* clearly labels deepest cortex in both species, but the stream of cells that subtends the mouse claustrum was not apparent in the macaque claustrum (Figures 7D,D′). *Synpr* was similar in claustral expression, yet general cortical expression was scattered in mouse while all layers were abundantly labeled in macaque (Figures 7C,C′). As mentioned above, *Rgs4* labels the cortex of both species, and reveals the macaque claustrum, without labeling that of the mouse (Figures 7E,E′). *Crym* and *Rorb* largely have negligible expression in the majority of both species' claustrums, but *Crym* reveals deepest cells in both (Figures 7G,G′) and *Rorb* reveals the putative superficial enndopiriform cluster of cells only in mouse and a gyral pocket of cells in the macaque inferior puddle (Figures 7H,H′). *Nr4a2* and *Ntng2* expression was similar between both species, both in claustral and overall cortical patterns (Figures 7A,A′,B,B′). These many variations were unexpected and suggest complex gene regulation that differs between species. Thus, the intriguing possibility for using the claustrum for the study of gene regulation emerges, with the concomitant inference that additional species need to be studied as well.

***Future studies***. This sampling of gene expression in the claustrum informs that both species and structural variation in labeling occur, but that to understand either, will require both more genes, and more claustrums. Within these two species, it appears that gene expression in the claustrum is mostly uniform, but selected examples show sufficient variation to establish regionalization important to consider when interpreting other data derived from other modalities. While it is tempting to suggest then that the claustrum could provide an anchor to study brain function based on its simple appearance and cortical affiliation, a more careful comparison will be needed first in order to establish this assertion, such as methods more quantitatively sensitive to gene or protein expression levels, and obviously more species and more areas within the claustrum.

However, one might use this limited knowledge to more quickly isolate exemplar genes and species that showcase conformity or derivation across a particular parameter. For example, online resources that “lump” all of the claustral components together spatially, may still suggest differential anatomical gene expression, by searching for expression levels that show more variation relative to other genes. This approach requires at least a few separate samples to provide possible variation, and the same reasoning could uncover genetic contributions based on known functional variation across species. Such approaches have probed mouse expression databases to find genes that mark cell types, such as Von Economo cells, thought only to occur in larger brains (Allman et al., 2011).

More careful studies, across modalities, would need to be pursued before redrawing of any current brain maps, and as always, borders should be regarded as ultimately probabilistic. However, the efficiency of taking publically available data to find exemplar genes that specifically mark the claustrum with those that suggest intraclaustral subdivision, and then probe other species and confirm and expand these results, is appealing in its simplicity.

It may be that the puddles are genetically and functionally homogenous, and thus make for prime targets of understanding brain/structure relationships. Or, it may be that mouse brains exhibit a vestigial claustrum. And it might be likely, that expanding the gene markers in non-human primates and human experiments would reveal subtle distinctions that underpin functional differences.

The null hypothesis in this case is that variation in claustral function does not reside in expression patterns of single genes, but that combinatorial patterns will need to be analyzed. Even then, it is unlikely that there are many genes that can exhibit an undue influence on a particular brain area. But those few make for powerful targets and the means of isolating them more efficient.

If the claustrum were molecularly homogenous, it would be unusual, and thus would provide a brain region where physiology and connectivity may be probed more accurately. With the claustrum as presently studied in typical laboratory species appearing mostly uniform, with some elaboration in larger brains, it is in that respect, like many other cortical brain regions. But any unusual uniformity remains to be shown, and gene markers are available to test predictions, and so some resolution could be forthcoming.

***A scenario***. The ideas presented by Reep (2000) led us to the following speculative line of thought about the specific direction for research. Layer 7, in those few taxa where it is seen, consists of remnants of the Subplate, that did not apoptose as subplate cells usually do, after they have initiated and supervised the organization of the cells migrating through the plate and forming the cerebral cortex. The presence of Layer 7 could signify a “half-baked” brain: finishing the cortex was abandoned in favor of shorter gestation period, quick reproduction, and reduced brain size and energy use. So the cortical details may have been left undone and since it did not interfere with survival, the layer persisted in its paedomorphic state.

The Subplate and Layer 7 are between the claustrum and the external capsule. Therefore, they might direct the puddling of claustrum along with the organization of the overlying cortex. The presence of layer 7 in rats and mice could mean that claustrum and puddles, as well as cortex, did not have a chance to completely develop. One undone feature may be the location of cortical cells scattered through the claustrum (Mathur et al., 2009) In the absence of Subplate instructions, their segregation might not have been accomplished.

Rats and mice belong to one of the few taxa where Layer 7 is found (Reep, 2000). Others include some bats and insectivores, all of whom could have profited by reducing brain size, weight, and energy cost. This raises questions about their suitability as models for the study of “complete” mammalian brains.

All of these questions need developmental studies to solve the riddles not only of claustrum, but also those of Layer 7 and the endopiriform regions. These developmental studies need proteomic analysis including that using gene expression, along with the array of other methods that can be used in large accumulations of specialized cells, including the advanced electrophysiological, neurochemical, and imaging techniques now available.

The Posterior Puddle of the Pig claustrum may be the optimal subject for such a comprehensive approach toward solution of all of these open questions. Along with its other advantages, the minipig brain has recently been shown to be suitable for transfection studies (Glud et al., 2011), and they have a suitably sized puddle for doing them.

### Conflict of interest statement

The authors declare that the research was conducted in the absence of any commercial or financial relationships that could be construed as a potential conflict of interest.
